# High Inter- and Intra- Diversity of Amino Acid Content and Protein Digestibility Disclosed in Five Cool Season Legume Species with a Growing Market Demand

**DOI:** 10.3390/foods12071383

**Published:** 2023-03-24

**Authors:** Elsa Mecha, Mara Lisa Alves, Andreia Bento da Silva, Ana Bárbara Pereira, Diego Rubiales, Maria Carlota Vaz Patto, Maria Rosário Bronze

**Affiliations:** 1 ITQB NOVA, Instituto de Tecnologia Química e Biológica António Xavier, Universidade Nova de Lisboa, Av. da República, 2780-157 Oeiras, Portugal; 2 iBET, Instituto de Biologia Experimental e Tecnológica, Av. da República, Apartado 12, 2781-901 Oeiras, Portugal; 3 Faculdade de Farmácia, Universidade de Lisboa, Av. das Forças Armadas, 1649-019 Lisboa, Portugal; 4 Institute for Sustainable Agriculture, CSIC, Avda Menéndez Pidal s/n, 14004 Córdoba, Spain

**Keywords:** protein, amino acids, protein digestibility, faba bean, chickpea, pea, lentils, grass pea

## Abstract

Legumes have been sought as alternative protein sources to ensure food security and environmental sustainability. Characterizing their protein content and quality, including in underutilized grain legumes, e.g., grass pea, gives value to the legumes’ underexplored variability. To fill the gap of knowledge in legumes’ protein quality, for the first time, five extensive collections of cool season grain legumes were cropped under the same environmental conditions and further analyzed. Multivariate analysis showed the existent intra- and inter-species variability. The legume species with the highest protein content, grass pea, *Lathyrus sativus* (LS), was not the one with the overall highest individual amino acids content and in vitro protein digestibility. With these last characteristics lentil, *Lens culinaris* (LC), was highlighted. The highest average values of arginine (Arg), glutamic acid (Glu), and threonine (Thr) were found in LS and *Vicia faba* (VF). *Cicer arietinum* (CA) stood out as the species with the highest values of Thr and methionine (Met). Regarding the in vitro protein digestibility (IVPD), LC, followed by *Pisum sativum* (PS) and LS, were the legume species with the highest values. Ultimately, this study bought to the fore legume species that are not commonly used in western diets but have high adaptability to the European agricultural systems.

## 1. Introduction

Finding the best/optimal dietary sources of protein for human and animal consumption with substantial cost savings and environmental benefits remains one of the major challenges of 21st-century modern societies, firmly rooted in ensuring food security for all [1]. To keep pace with the expected world population growth from 7.5 billion to 9–10 billion people by 2050 [2], alternative dietary protein sources with lower environmental impact (lower contribution to greenhouse gas emissions, deforestation, and water consumption) must be explored [3].

Grain legumes are environmentally friendly, sustainable sources of protein [4] due to their positive impact on soil quality through symbiotic nitrogen fixation, carbon sequestration, nutrients, and water retention. Cropping legumes in a diversity of systems also contributes to reducing the emission of greenhouse gases (CO_2_ and N_2_O) and improves weed, pest, and diseases control. Overall, the inclusion of legumes in cropping systems, contributes to increasing crops’ productivity, especially in regions, including European countries, where affordable plant protein production is of utmost importance for human and animal food security [5,6]. Additionally, grain legumes, by virtue of their nutritional and bioactive composition, have been related to chronic disease prevention (e.g., diabetes *mellitus* type 2, cardiovascular diseases, and colon cancer) [7].

Despite the several advantages for the food and feed systems, legume cultivation, especially in Europe, has been in drastic decreasing, consequence of the annual yield fluctuations due to susceptibility to biotic and abiotic stresses, high phosphorus requirement, and relatively low profitability [8]. Nevertheless, the demand for locally produced legume protein for human consumption and feeding purposes [9] increased, and with it, a reborn interest in grain legume production. Until now, most of the plant protein supply in the European market has been relying on imported soybean. Indeed, protein is the costliest nutrient in animal feed formulations [10]. Despite the different animal specificities in protein and amino acid requirements, most terrestrial animals require 20% protein in the feed formulations, and in carnivorous species, such as cats, the required protein can easily reach 38%. In aquaculture, the protein requirements for fish production in the formulated diets can reach values of 50% [10].

Aside from the promotion of local legume production, the diversification of protein sources in human and animal diets and the increase in the European consumption of grain legume protein are imperative to minimize the health and environmental risks linked to an animal-based protein diet. Under this scenario, the protein requirements in humans and animals should attempt dietary shifts towards less animal-based and more plant-based food products, focusing mainly on local production to ensure competitive production prices [5]. Moreover, legumes are an excellent source of cheap, sustainable high protein content and are rich in the essential amino acid lysine and poor in the thiol-containing amino acids (methionine and cysteine), being easily complemented by the cereals’ protein to achieve a balanced consumption of amino acids. Defined by the bioavailability of essential amino acids, which is intrinsically related to the protein’s amino acid composition and protein digestibility [11], the grain legumes’ dietary protein quality may depend on the legume species and varieties under study.

To better promote grain legume production and consumption, a detailed study of the nutritional makeup is required to take full advantage of each species’ nutritional potential. A comprehensive biodiversity characterization of the different traits influencing protein quality (amino acids profiles, anti-nutrient factors, and digestibility scores) is still not available, at least for most of the potential cool season grain legumes, under the European agricultural systems. The available data on protein content [12] do not highlight the existent intra-diversity within legume species, and, so far, there is no comprehensive composition database on their protein quality that emphasizes grain legumes’ intra- and inter-diversity. Moreover, the data regarding the protein content and quality of neglected or under-utilized grain legumes, such as grass pea (*Lathyrus sativus*), are still missing. These species can be fundamental to mitigating hunger and population protein deficit under adverse climate conditions, such as arid and hot environments [6]. As a working hypothesis, the different cool season legume species produced under the European scenario have natural variability not only in the protein quantity but also in the protein quality. This variability can be exploited through analytical methodologies and compiled for further application in legumes’ protein improvement. To fill the existent gap of knowledge, the present study aims to explore the inter and intra-variability of protein content and quality (measured by amino acids composition and in vitro protein digestibility) on extensive collections of five of the most important culinary cool season grain legumes, traditionally produced and consumed in European countries, including faba bean, *Vicia faba* L., pea, *Pisum sativum* L., lentil, *Lens culinaris* L., chickpea, *Cicer arietinum* L., and grass pea, *Lathyrus sativus* L., and their few crop wild relatives, with around 100 genotypes each, representative of the existent diversity, and with potential interest for the European legume breeders. The knowledge generated under this study will allow a clearer picture of the overall protein content and quality variability within and among these five grain legumes species, creating new opportunities to address protein deficiencies by incorporating grain legumes into nutritional and agriculture policies and, when needed, in the development/breeding of higher quality varieties, answering to sustainability issues.

## 2. Materials and Methods

### 2.1. Plant Material

Five different collections of cool season grain legume species, *Cicer arietinum*—CA, *Lens culinaris*—LC, *Lathyrus sativus* and their crop wild relatives—LS, *Pisum sativum* and their crop wild relatives—PS, and *Vicia faba*—VF, accessions were cropped with autumn–winter sowing, under the same field conditions in Córdoba, Spain (Global Positioning System, GPS, coordinates: latitude–37°53′29.58″ N and longitude–4°46′21.90″ W). Plants were grown following standard practices in the area, planted in December under rainfed conditions, and hand weeded as needed. Harvest was manual, and the seeds were stored at 5 °C until analysis. The analyzed accessions from each legume species collection (CA, *n* = 86; LC, *n* = 92; LS, *n* = 109 of which two were crop wild relatives accessions of *Lathyrus cicera*; PS, *n* = 118 of which two were crop wild relatives accessions of *Pisum fulvum*, one of *P. abyssinicum*, three of *P. sativum* ssp. *arvense*, one of *P. sativum* ssp. *elatius*, and one of *P. sativum* ssp. *syriacum*; VF, *n* = 92) were selected from germplasm breeding collections at IAS-CSIC and IFAPA Alameda del Obispo, or from different plant germplasm banks (CRF, Madrid; INIAV-Oeiras, PRT005; USDA, USA; and ICARDA). The selection comprised crop wild relatives, landraces, cultivars, breeding lines, and commercial varieties (Table S1), allowing us to study not only the inter-species but also the intra-species variability within each legume species.

### 2.2. Chemicals and Reagents

A Millipore–Direct Q3 UV System (Molsheim, France) was used to obtain Milli-Q^®^ water (18.2 MΩ·cm). For the HPLC analysis, the reagents Chloride acid (HCl), formic acid (98% *p.a*), and acetonitrile HPLC gradient grade were obtained from Carlo Erba Reagents SAS (Val de Reuil, France). Amino acids standard H was purchased from Thermo Fisher Scientific (Rockford, MA, USA), and the phenol BioXtra ≥ 99.5% (GC), nonafluoropentanoic acid, bovine trypsin from bovine pancreas ≥ 10,000 BAEE units/mg protein, calcium chloride dehydrated (CaCl_2_·2H_2_O), benzoyl-L-arginine-p-nitroanilide (L-BAPA), dimethyl sulfoxide (DMSO), tris(hydroxymethyl)aminomethane, glacial acetic acid (≥99%), sodium hydroxide (NaOH), porcine trypsin type IX-S, bovine α-chymotrypsin, type II, *Streptomyces griseus* protease, type XIV and casein from bovine milk were purchased from Sigma-Aldrich (St. Louis, MI, USA).

### 2.3. Samples Preparation

The mature dried seeds from each accession of the different species were milled (Falling n◦ 3100–Perten, Hägersten, Sweden), sieved to 0.8 mm, and stored at −20 °C.

### 2.4. Total Protein

As described in Serrano et al., 2017 [13], Santos et al., 2018 and 2019 [14,15], the total protein (%) was assessed by near-infrared (NIR) analysis (MPA; Bruker, Billerica, MA, USA), with flour calibrations for grain legumes provided by Bruker (*n* > 500; *R*^2^ > 90). The NIR data were validated with 10% of the selected samples to cover the range and characterized by the reference method, which, in the case of the protein content, was the combustion method, ISO 16634-2:2016 [16]. The total protein was determined by converting nitrogen concentration with a factor of 6.25 [13].

### 2.5. Protein Quality

#### 2.5.1. Amino Acids’ Extraction

Amino acids were extracted in duplicate following the previously described procedure [17]. During a 6 h acidic hydrolysis at 150 °C, 0.5 g of legumes’ seed whole flour were kept in contact with a chloride acid solution of 6 M and 0.1% phenol (7 mL). After concentrating until dryness, in a Speedvac concentrator (Labconco^®^, Kansas City, MO, USA), a solution of HCl 0.1 M (5 mL) was used to reconstitute the volume. After centrifugation at 5000× *g* for 15 min, the supernatant was collected, filtered through 0.20 µm cellulose acetate filters, and frozen at −20 °C, for further analysis.

#### 2.5.2. Amino Acids’ Content

For the amino acids’ quantitation, an HPLC-MS/MS system from Waters Alliance 2695 HPLC system coupled to a triple quadrupole mass spectrometer, Micromass^®^ Quattro micro-API (Micromass, Waters, Milford, MA, USA), equipped with an electrospray ionization source (ESI) was used. The chromatographic separation was performed with a Mediterranean Sea 18, 5 µm, 20 × 0.21 cm, 1.8 µm, (Teknokroma^®^, Barcelona, Spain) column at 45 °C. The eluents, an aqueous solution of 0.1% formic acid with 0.15% of nonafluoropentanoic acid (eluent A) and an acetonitrile solution of 0.1% formic acid with 0.15% of nonafluoropentanoic acid (eluent B), were used in a gradient mode during 45 min at a flow rate of 0.3 mL/min. The gradient elution started with 2% of eluent B and was kept at this concentration for three minutes. Then, the percentage of eluent B increased to 25% in 22 min, remaining at this concentration for two minutes. The initial conditions, 2% of eluent B, were re-established in 18 min. The temperature of the ionization source was programmed at 130 °C with a cone voltage of 20.0 V and capillary voltage of 2.70 kV. Nitrogen (N_2_) was used as a drying and nebulizing gas, and Argon (Ar) as the collision gas. A dilution of the amino acids’ extracts (1:1000) in eluent A was made, and the diluted extracts were maintained at 10 °C until injection. The sample injection volume was 20.0 µL. A total of 16 amino acids (Serine, Ser; Aspartic acid, Asp; glycine, Gly; glutamic acid, Glu; threonine, Thr; alanine, Ala; proline, Pro; valine, Val; methionine, Met; histidine, His; tyrosine, Tyr; lysine, Lys; isoleucine, Ile; leucine, Leu; arginine, Arg; phenylalanine, Phe) were analyzed in ESI positive mode by multiple reaction monitoring (MRM) modes, following the experimental conditions described elsewhere [18]. The amino acids’ identification was performed by comparison with the standards retention time and *m*/*z* values, and the quantitation was made by calibration curves prepared in eluent A with different concentrations (3.8–30 µM). The data were acquired and processed using MassLynx software, version 4.1 (Waters, Milford, MA, USA). The results were presented in g/16 g of nitrogen (N), as well as in g/100 g of sample.

#### 2.5.3. In vitro Protein Digestibility

The in vitro protein digestibility (IVPD) was conducted following the pH-drop procedure proposed by Tinus et al., 2012 [19]. Briefly, the equivalent of 62.5 mg of protein was weighed in legume raw whole flour and added to 10 mL of Milli-Q^®^ water. The mixture was incubated at 37 °C and stirred with a magnetic bar for 1 h. The started pH was adjusted to 8.0 with a solution of NaOH and/or HCl (0.1 M). After preparing, on the analysis day, a multi-enzyme solution (10 mL) of porcine trypsin (16 mg), bovine chymotrypsin (31 mg), and *Streptomyces griseus* protease (13 mg), maintained at 37 °C, the pH was also adjusted to 8.0. The pH drop was measured in a pH meter Metrohm 703 Ti Stand with stirrer and pump (Metrohm, Herisau, Switzerland) every five seconds for 15 min after adding 1 mL of the multi-enzyme solution to the sample. Bovine casein was used as the control for in vitro digestibility comparison. Considering the limited amount of sample, the analyses were performed in duplicate for two different varieties, selected by convenience, from each legume species. The in vitro protein digestibility (IVPD%) was determined according to the equation (Equation (1)):

IVPD (%) = 65.66 + 18.10 × (∆pH 10 min)(1)

#### 2.5.4. Calculated Protein Quality

The amino acid score of the essential amino acids (EAAs), only obtained from the diet, was determined by comparison with the recommendations for 2 to 5 years old children. This comparison followed the FAO/WHO experts’ recommendation regarding the use of amino acid requirements of children between 2 to 5 years old to assess the protein quality of foods for all the age groups above 2 years old [20]. The amino acid with the lowest score was defined as the limiting amino acid [21]. For the protein efficiency ratios (PER), Equations (2)–(4) [22] were used.

PER 1 = −0.684 + 0.456 × Leu − 0.047 × Pro(2)

PER 2 = −0.468 + 0.454 × Leu − 0.105 × Tyr(3)

PER 3 = −1.816 + 0.435 × Met + 0.78 × Leu + 0.211 × Hys − 0.944 × Tyr(4)

For the in vitro protein digestibility corrected amino acid score (IVPDCAAS), Equation (5) was applied:

IVPDCAAS score = Lowest amino acid score × IVPD(5)

### 2.6. Statistical Analyses

Descriptive statistics, mean, and standard deviation for each trait were accessed for all species. Histograms and boxplots were used to visually access data distribution. Outlier accessions were removed from the analysis. Assumptions of normality (Shapiro–Wilk’s test) and variance homoscedasticity (Levene’s test) were analyzed, assuming a confidence interval of 95%. Whenever necessary, Box–Cox transformation was applied to ensure the normality of the residuals. The traits whose values were transformed were re-coded by adding the suffix “Tran” to the initial code label. Univariate analysis of variance was conducted by one-way ANOVA followed by Tukey–Kramer multiple comparison test (at a significance level of 5%). Pearson’s pairwise correlations were established between the amino acids and the total protein contents for all and each legume species. A scatter plot matrix was used to summarize the data distribution and the pairwise correlations in a single representation. Multivariate principal component analysis (PCA) was applied to the standardized data, considering the total protein and the amino acid contents of the different legume accessions to visually determine potential relationships between species or accessions within the different species and the measured traits. The number of retained components was based on the Kaiser criterion (retaining the components with eigenvalues higher than one). To maximize the differences between legume species, the canonical variate analysis (CVA) was applied, using the Mahalanobis distances to establish the inter-species means. Data analysis was performed using the GenStat software (GenStat^®^ for Windows 19^th^ Edition, VSN International Ltd., Hemel Hempstead, UK) [23].

## 3. Results and Discussion

To valorize the local production and consumption of grain legumes in the European context, a detailed study on the European most important culinary cool season grain legumes’ protein content and quality, measured by amino acids composition and in vitro protein digestibility, was undertaken. Five representative collections of faba bean, pea, lentil, chickpea, and grass pea accessions and a few wild relative crops, with potential interest for the European legume breeders, were cultivated under the same environment and comparatively analyzed to take full advantage of the nutritional potential of each species. This analysis provided meaningful information, freely accessible to breeders, food scientists, and farmers to help them avoid high costs with imported feeding materials and to keep up with the goal of a sustainable diet for humans and animals that matches physiological needs with minimal excretion or waste losses.

### 3.1. High Diversity Detected in Protein Content and Quality among but Also within Five Cool Season Grain Legume Species

**Figure 1:**
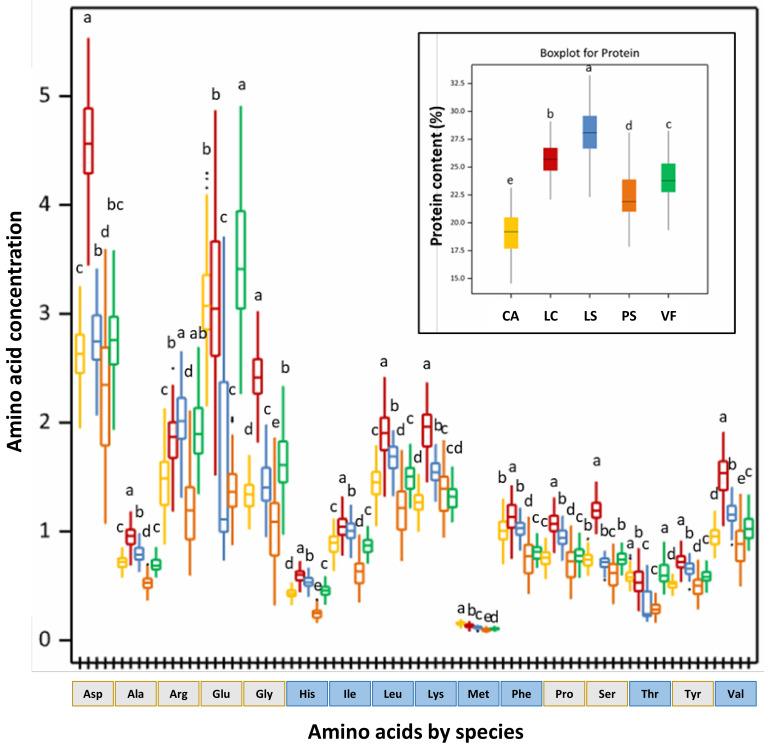
Box plots comparing the results obtained for protein quantity and quality from five grain legume species. Above each box plot, the letters correspond to the result of the species means comparison using a Tukey–Kramer multiple comparisons test; levels that are not significantly different from each other are represented with the same letter. Legend: in yellow, *Cicer arietinum* (CA), *n* = 86; in red, *Lens culinaris* (LC), *n* = 92; in blue, *Lathyrus sativus* and their crop wild relatives (LS), *n* = 109; in orange, *Pisum sativum* and their crop wild relatives (PS), *n* = 118; and in green, *Vicia faba* (VF), *n* = 92. Traits’ legend: Ala—Alanine, Arg—Arginine, Asp—Aspartic acid, Glu—Glutamic acid, Gly—Glycine, His—Histidine, Ile—Isoleucine, Leu—Leucine, Lys—Lysine, MetTran—Methionine transformed values, PheTran—Phenylalanine transformed values, Pro—Proline, Protein—Protein content, Ser—Serine, Thr—Threonine, Tyr—Tyrosine, and Val—Valine. The traits with a blue color represent essential amino acids.

As shown in Figure 1 and Table S2, the protein and the amino acid contents distribution in the different legume species were quite variable. Although less utilized than the other four analyzed species, LS stood out as the grain legume species with the highest average protein content (28.17 ± 1.97 g/100 g) and wider intra-species variation, followed by LC, VF, PS, and CA species. In fact, the grass pea has been highlighted as one of the grain legumes with the highest protein content showing, for Indian LS cultivars (Prateek and LP-24) mature seeds, protein values between 33.26 and 39.24% [24]. Aside from the high protein content in LS seeds, a wide intra-variability, with values ranging between 8.6% and 32.2%, was previously reported for 40 genotypes of Indian LS and attributed to genetic and environmental factors [25,26]. In addition to these factors, differences in the analytical methodologies applied for the protein analysis (Kjeldahl method from the Association of Official Analytical Chemists, AOAC, Dumas’s combustion method to determine nitrogen percentage, or near infrared spectroscopy, NIR) may impair data comparison among different studies, as revised for the lentils protein content [27]. In the present study, the average protein content determined in the lentil accessions, 23.97 ± 1.89 g/100 g, was slightly lower than the average value determined in 361 lentil samples collected from the Crop Development Centre (CDC) at the University of Saskatchewan, Canada, 29.64 ± 1.65 g/100 g [28] but within the range of values determined in four LC varieties grown in Romania, 25.6–28.9 g/100 g [29]. The low average value obtained for the chickpea protein content (19.08 ± 2.03 g/100 g) was in accordance with the reported amounts, 16.1–26.7, and 19.9–25.5 g/100 g, for different chickpea seed types, “desi” vs. “kabuli”, respectively [30]. PS, with an average protein content of 22.47 ± 2.63 g/100 g in the present study, was within the range of values described for PS varieties, 21.17–24.90 g/100 g [31] and in agreement with the range of values determined for the commercial pea varieties, AC Agassiz (21.8 ± 0.8–35.7 ± 1.7%) and CDC Saffron (21.7 ± 1.0–30.1 ± 0.8%) [32]. In this last study, the measurement of ten pea seeds from each variety, characterized by different sizes, suggested that within each variety, the seed size can be a relevant factor for protein content variability. For the VF seeds, the average protein content in the present study, 23.97 ± 1.89%, was slightly lower than the described values for eleven faba bean varieties grown in Canada (27.5 to 32.4%). Differences in the genotype and environmental conditions might have contributed to such differences [33].

In general, for the five different legume species under study, the three most abundant and variable amino acids were Arg, Glu, and Asp, and the three less abundant and variable were Met, Thr, and His. Overall, the amino acid concentrations and ranges changed in a species-specific way, with the few crops’ wild relatives under study showing similar concentrations as the respective grain legume species crop. The crop wild relatives group of *Lathyrus sativus*, in our study, composed of accessions of *L. cicera*, shared with some grass pea accessions with similar protein content and quality. For the crop wild relatives of pea, such as accessions from *P. fulvum*, *P. abyssinicum*, *P. sativum* ssp. *arvense*, *P. sativum* ssp. *elatius*, and *P. sativum* ssp. *syriacum*, the quantity and quality protein pattern were similar to the remaining pea accessions.

In CA and VF, the three more abundant amino acids were, by descending order, Glu > Asp > Arg; in PS and LC, Asp > Glu > Arg and LS, Asp > Arg > Glu. If for Met, a limiting sulfur-containing amino acid in legumes, the intra-species variability was reduced, with average contents quite narrow in all the studied collections of different legume species, ranging between 0.10 ± 0.01 g/100 g (PS) and 0.15 ± 0.02 g/100 g (CA), for Asp and Glu the values were more disperse, varying, for Asp, between 4.49 ± 0.54 g/100 g (LC) and 2.30 ± 0.64 g/100 g (PS), and for Glu, between 3.51 ± 0.59 g/100 g (VF) and 1.44 ± 0.31 g/100 g (PS).

LS was the studied legume species with the wider variation in the essential amino acid, Thr (52.0%), and in the non-essential amino acid, Glu (59.1%), indicating high diversity and, so, high potential for breeding. For this variability, the crop wild relatives *L. cicera* (*n* = 2) accessions made no significant contribution. LC depicted the highest average values for most of the quantified amino acids. The few exceptions included the Met, Thr, Arg, and Glu.

For these amino acids, LC average contents were surpassed by the CA (Met and Thr), LS (Arg), and VF (Glu) legume species, see Table S2. Compared with the previously reported values for LC varieties [29], the results described herein for Lys (1.89 ± 0.27 g/100 g), Leu (1.87 ± 0.26 g/100 g), Phe (1.13 ± 0.15 g/100 g), Tyr (0.70 ± 0.10 g/100 g), and Ser (1.18 ± 0.19 g/100 g) were in agreement with the reported values in four LC Romania varieties (1.82–2.03 g/100 g, Lys; 1.59–2.14 g/100 g, Leu; 1.07–1.52 g/100 g, Phe; 0.62–0.72 g/100 g, Tyr; and 0.06–1.49 g/100 g, Ser). Except for Lys (VF), and Tyr (CA), most of the amino acids in PS showed the smallest average values among the five legume species. On average, the determined values for Asp (10.20 ± 2.36 g/16 g N), Gly (4.62 ± 1.48 g/16 g N), Arg (5.51 ± 1.47 g/16 g N), His (1.09 ± 0.18 g/16 g N), Val (3.92 ± 0.78 g/16 g N), and Ile (2.82 ± 0.54 g/16 g N) were in accordance with the previously described results for seven pea cultivars, Ucero, Ramrod, Agra, Terno, Xantos, Suit, and Achat, (9.98–10.69 g/16 g N, Asp; 3.92–8.26 g/16 g N, Gly; 4.12–9.68 g/16 g N, Arg; 1.03–2.22 g/16 g N, His; 3.61–4.72 g/16 g N, Val and 2.52–4.23 g/16 g N, Ile) [31]. VF seeds with potential interest for animal (livestock) nutrition, as a substitute for soybean [34], revealed high contents of Glu (14.66 ± 2.24 g/16 g N) and Arg (8.14 ± 0.86 g/16 g N), in line with the reported Glu (12.50–18.79 g/16 g N), and Arg (5.21–12.10 g/16 g N) contents determined in different Swedish faba bean cultivars [35]. These amino acids are key players in human and animal health maintenance, acting as functional amino acids and supporting milk, meat, and/or egg production [36,37,38]. The arginine and the homoarginine produced enzymatically, from arginine and lysine, by AGAT (arginine: glycine amidinotransferase) activity have been negatively associated with adverse cardiovascular events and cardiovascular mortality [39]. Although the protein characterization was performed in just one environment, the considerable variability detected among the studied traits in Córdoba highlights the genetic richness of the studied grain legume collections and at least their potential for specific local breeding [17,40].

**Figure 2:**
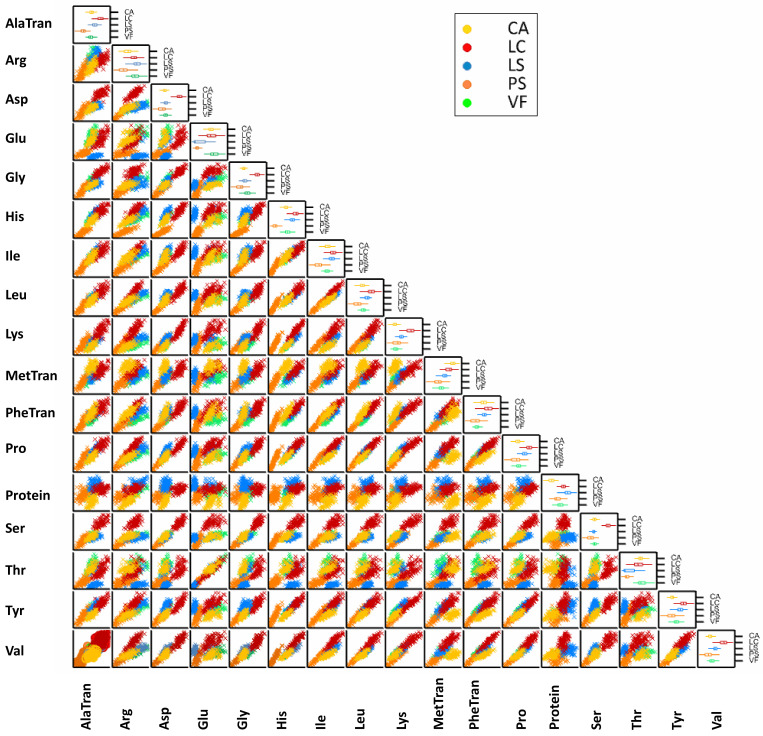
Scatter plot matrix showing the pairwise relationships between traits and in the diagonal, the box plots showing the data trait distribution for each species. Legend: in yellow, *Cicer arietinum* (CA), *n* = 86; in red, *Lens culinaris* (LC), *n* = 92; in blue, *Lathyrus sativus* and their crop wild relatives (LS), *n* = 109; in orange, *Pisum sativum* and their crop wild relatives (PS), *n* = 118; and in green, *Vicia faba* (VF), *n* = 92. Traits’ legend: AlaTran—Alanine transformed values, Arg—Arginine, Asp—Aspartic acid, Glu—Glutamic acid, Gly—Glycine, His—Histidine, Ile—Isoleucine, Leu—Leucine, Lys—Lysine, MetTran—Methionine transformed values, PheTran—Phenylalanine transformed values, Pro—Proline, Protein—Protein content, Ser—Serine, Thr—Threonine, Tyr—Tyrosine, and Val—Valine.

Regarding the pairwise correlations between the individual amino acids and between the amino acids and the protein contents, as summarized in the scatter plot matrix, Figure 2, with few exceptions, such as in CA species, there were strong correlations between the protein content and Arg, Asp, His, and Phe, Pearson’s *R* of 0.854, 0.818, 0.700, and 0.702, respectively. The weak correlations (Pearson’s *R* < 0.5) between the protein and the amino acids content in the different legume species indicated that the total protein content, determined by NIR, should not be used as a predictor of the individual amino acid content. The correlations between protein content and the different amino acids under study also varied in a species-specific way. If for the pair Thr × Glu, in the different legume species, the Pearson’s *R* ranged only between 0.874 (CA) and 0.994 (LS), for the other amino acids pairs, such as Pro × Glu, the positive correlation coefficients were much more variable among the studied grain legumes, ranging from 0.187, in LS, to 0.729, in LC. This difference could be attributed to inter-species diversity in the amino acids’ pathways. Glu, for instance, can be involved not only in Pro synthesis but also in the alternative pathway of Arg synthesis. Glu is a central amino acid in plant growth and development [41], participating directly in the biosynthesis of proline by pyrroline-5-carboxylate in the plant cells cytoplasm or being directly synthesized from Pro by the activity of 1-pyrroline-5-carboxylate dehydrogenase [41]. In the chloroplasts of the plant cells, Glu can also be involved in the biosynthesis of Arg through the action of the ornithine aminotransferase in the ornithine synthesis pathway. The positive strong correlation between Pro and Arg is quite evident in Figure 2, for all the legume species, with correlation coefficients ranging from 0.783, in LS, to 0.949, in LC. Ornithine cyclodeaminase is responsible for converting Arg to Pro in an alternative pathway for Pro biosynthesis [42]. Arginine is a precursor of nitric oxide (NO), a free radical, and polyamines (e.g., putrescine, cadaverine, and spermidine), which play a key role in responses to biotic and abiotic stresses in higher plants such as legume plants, contributing for their survival under challenging environments as effective agents against pathogenic bacteria, fungi, and parasites [42]. The shared pathway between Ala and Asp (synthesized by the transamination of oxaloacetate and pyruvate [43] explains the positive strong correlation between these amino acids in the different legume species (Pearson’s *R* varying from 0.772 for LC to 0.844 for PS). The similarity of the amino acids’ nature [43], such as Ile, Leu, and Val, as non-polar branched-chain amino acids, could contribute to explaining the high correlation coefficients between these amino acids in particular (Pearson’s *R* correlation: 0.854 in LS and 0.986 in PS, for the pairwise correlation between Leu and Ile, and Pearson’s *R* correlation 0.866 in VF and 0.986 in PS, for the pairwise correlation between Ile and Val). Thr was moderately to highly correlated to Gly in all the studied legume species (Pearson’s *R* 0.633 in CA and 0.761 in PS). Such correlation can be attributed to the shared metabolic pathway between Thr and Gly, catalyzed by threonine aldolase, which participates in the Gly biosynthesis from Thr [44]. Although little is known about the His metabolism [45], His biosynthesis requires 5′-phosphoribosyl-1-pyrophosphate (PRPP), which is a key intermediate in the interconversion of proline, ornithine, and glutamate [46]. In this study, the high correlation coefficient between Lys and His in all the studied legume species (Pearson’s *R* 0.935 in LS and 0.966 in PS) could be explained by this shared metabolic pathway. Lys was also highly correlated to Met (Pearson’s *R* correlation 0.644 in CA and 0.933 in PS), in consequence of the bidirectional pathway synthesis that shares the precursor aspartate 4-semilaldehyde [47].

As shown in Table 1, regarding the calculated protein quality parameters, CA and PS were, respectively, the legume species with the highest and lowest amino acid scores and protein efficiency ratios (PER), indicating that with the exception of Met, the remaining amino acids were fully supplied by the CA species, and in agreement to the amino acids’ pre-school children (2–5 years old) requirements. For the other legume species, not only Met but also Thr, and in PS, also His, were below the requirements (less than 90% of the requirements). These results clearly showed the diversity in the legume species protein quantity and quality and highlighted the relevance of analyzing not only the amount of protein but also the amino acids composition for fair conclusions regarding the supply of good quality protein.

Aside from the nutritional value, the unique composition of free amino acids in plant-derived peptides is also related to the strong antioxidant activity exerted by some amino acid residues (e.g., Tyr, Met, His, Lys, Pro) as metal-ion chelators, active oxygen-quenchers, and free radical scavengers [48]. Therefore, the legume species, such as CA and LC, with higher amounts of the previous amino acids per gram of protein, and consequently with a higher amino acid score for the essential amino acids, are expected to have a higher rate of functional peptides with potential beneficial health effects, such as antimicrobial and antihypertensive effects [49,50].

Notwithstanding the diversity in the protein quality, promoted by differences in the amino acids content, the in vitro protein digestibility of the different legume species was also variable (sorted by descending order of the IVPD values, LC > PS > LS > CA > VF), ranging between 70.54% (VF) and 73.13% (LC). The determined IVPD values were slightly lower than the ones determined for the Canadian varieties of red and green lentils (84.24% and 83.28%, respectively), green and yellow peas (84.85% and 85.09%, respectively), chickpea (78.93%) and faba beans (81.77%) [51]. Although static and based on multi-enzyme systems, the pH drop procedure for the protein in vitro digestibility has been validated by comparison with the in vivo protein digestibility in rats, with a correlation coefficient of 0.93 [52], suggesting the potential of the in vitro methods to predict the protein true digestibility. Nevertheless, for the analysis of complex samples, the in vitro protein digestibility determined by the pH static method was considered to be low accuracy and a laborious methodology; therefore, other approaches could be adopted. An example is the in vitro protein digestibility static model proposed by INFOGEST that seeks to mimic the three phases of gastrointestinal digestion, oral, gastric, and intestinal accurately, reproducing the physiological parameters during the digestion process, such as the pH values, the temperature, the enzymatic concentrations, the duration of the digestion process, and the salt concentration [53].

The presence of secondary metabolites, such as phenolic compounds, phytic acid, and enzymatic inhibitors, the accumulation of β-sheet and α-helix protein secondary structure [54], and the predominance of globular proteins that fold into densely packed structures are responsible for proteins less prone to digestibility [55], determining much lower values of protein digestibility in legumes (around 75%) than the ones determined for the animal protein (90%) [56]. As previously mentioned, [54] despite the differences between the vegetable and the animal protein digestibility, the IVPD values in legume flours are expected to increase after applying traditional (e.g., cooking, extrusion, germination, and fermentation) or emerging processing methods (e.g., high-pressure processing (HPP), ultrasound, irradiation, pulsed electric field (PEF), and microwave). This is in consequence of the secondary metabolites decrease (e.g., phenolic compounds, phytic acid, saponins, and trypsin inhibitors). However, this is not a cost-effective solution for animal feeding. Therefore, characterizing the nutritional and anti-nutritional composition of the different legume varieties within each legume collection paves the way towards varieties with lower levels of anti-nutritional compounds and, consequently, with higher digestibility, more adequate for livestock nutrition.

### 3.2. Integrating the Protein Diversity in the Legume World—Featuring the Differences

**Figure 3:**
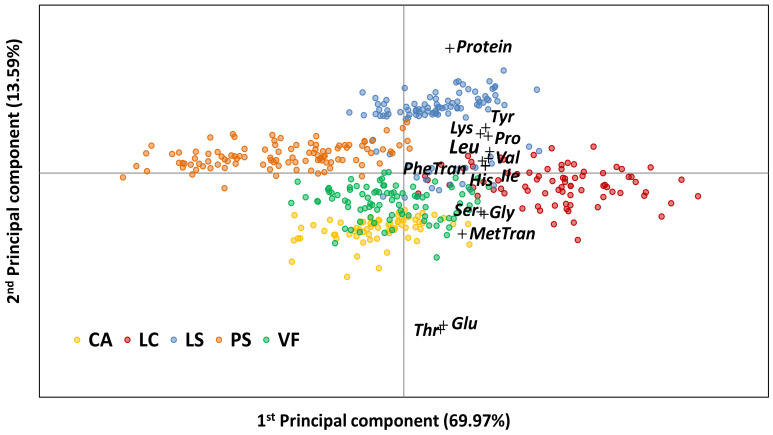
Principal component analysis biplot based on 17 traits related to protein quantity and quality in five grain legume species. Legend: in yellow, *Cicer arietinum* (CA), *n* = 86; in red, *Lens culinaris* (LC), *n* = 92; in blue, *Lathyrus sativus* and their crop wild relatives (LS), *n* = 109; in orange, *Pisum sativum* and their crop wild relatives (PS), *n* = 118; and in green, *Vicia faba* (VF), *n* = 92. The variance explained by each principal component is given in the axis heading.

**Figure 4:**
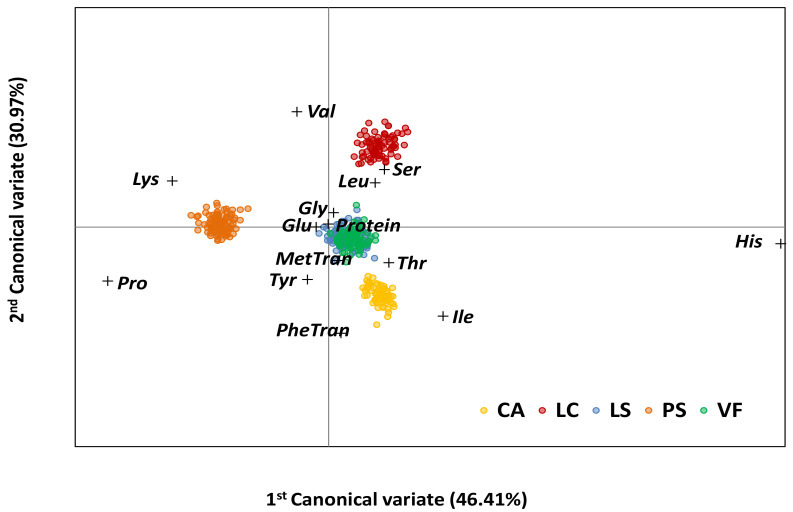
Canonical variate analysis biplot based on 17 traits related to protein quantity and quality in five grain legume species. Legend: in yellow, *Cicer arietinum* (CA), *n* = 86; in red, *Lens culinaris* (LC), *n* = 92; in blue, *Lathyrus sativus* and their crop wild relatives (LS), *n* = 109; in orange, *Pisum sativum* and their crop wild relatives (PS), *n* = 118; and in green, *Vicia faba* (VF), *n* = 92. The variance explained by each canonical variate axis is given in the axis heading.

Two different multivariate data analysis approaches were applied to integrate all the collected data: the unsupervised principal component analysis (PCA), Figure 3, and the supervised canonical variate analysis (CVA), Figure 4. If the former summarizes the spatial distribution of the different accessions, focusing on finding the combinations that account for most of the variance within the dataset, the latter amplifies the differences between the legume species and analyses the groups’ structure, focusing on finding combinations that maximize the differences between two or more datasets [57].

As shown in Figure 3, the distribution of the analyzed legume accessions in a space defined by the two first principal components retains 83.56% of the total explained variance. These two components separated each legume species and the respective crop wild relative’s collection, with a certain overlap between the VF and CA groups or between the smallest LS subgroup, located at a lower position in the PCA score plot, with the PS, VF, and LC collections. Based on the correlation coefficients of the different studied traits (protein and individual amino acids contents) and the defined principal components, Table S3, the protein content, as well as the Glu and Thr contents, mostly related to PC2, were responsible for sample dispersion along the vertical axis of PC2. The other traits were mostly associated with sample dispersion along the horizontal PC1 axis. In fact, the superimposition of the different legume species groups in the PCA representation is in agreement with the descriptive analysis performed in the previous sections of this discussion. A clear separation is observed between the largest LS subgroup (in the upper position of the PCA score plot), the VF, and the CA species (in the bottom) arranged in opposite positions along the vertical PC2 axis. This is related to the protein content, higher in the LS and lower in the VF and CA species, but also to the Glu and Thr amino acid contents, lower in LS but higher in VF and CA, respectively. The high variability within the different legume species was clear in the PCA analysis, particularly in the LS species, with a distinct separation along the PC2 axis into two different LS subgroups (Table S4). While the upper subgroup of LS samples (*n* = 80) showed higher protein content (28.36 ± 1.79 g/100 g) and lower Gly (1.37 ± 0.16 g/100 g), Glu (1.05 ± 0.13 g/100 g) and Thr (0.23 ± 0.02 g/100 g) contents, the lower subgroup of LS samples (*n* = 24), which included the crop wild relative accessions of *L. cicera*, was characterized by slightly lower protein content (27.62 ± 2.40 g/100 g) but higher Gly (1.71 ± 0.16 g/100 g), Glu (3.05 ± 0.35 g/100 g), and Thr (0.59 ± 0.07 g/100 g) contents. In the upper subgroup of LS samples, most of the accessions had Mediterranean (37.5% of the samples) or South Asian origins (31.3%) with a predominance of small size and dark seeds (80% and 58.8%, respectively). In the lower subgroup, 52.4% of the LS samples had a Mediterranean origin, with heterogeneity in the size and color of the seed (Table S3). The diversity in the protein content and especially in the accessions’ amino acid composition, allied with the LS subgroup’s diversity on the accessions’ geographical origin, seed size, and color, represent an opportunity to improve the LS protein content and quality in any of the LS geographical/ morphological groups.

Along the horizontal PC1 axis, the remaining amino acids are responsible for the variability and opposite position occupied by the PS and LC accessions, which were generally characterized by the lowest and highest individual amino acid contents, respectively.

The CVA analysis in Figure 4, supported by the inter-species Mahalanobis distances, indicates that the first two canonical variates retained more than 75% of the total explained variance (77.38%). In Figure 4, the most distant and well-separated legume species in the space defined by the two first canonical variates were CA, LC, and PS. In the middle of the three previous legume species (CA, LC, and PS), LS and VF occupied a central position in the CVA analysis. The lowest His content in the PS legume species, compared to the other legume species, contributed to the occupied isolated position of this legume species in the left corner of Figure 4. The higher Thr content in CA and VF species, *p* < 0.05, compared to the other analyzed legume species, their similar contents in Val, Leu, Ile, and Ser, and the high Phe content in CA, comparable to the one in LS species, justify the framing of the CA samples to the bottom and right position on the CVA plot, Figure 4. The overall individual amino acids’ higher contents (e.g., Val, His, Leu, and Ser) in the LC legume species explains its position on the top and right corner of the space defined by the two first canonical variates.

## 4. Conclusions

Focused on exploring the protein content and quality of five collections of distinct grain legume species cropped under the same environment, the present study showed, for the first time, the existent diversity among and within the representative collections of faba bean, lentil, chickpea, grass pea, and pea European breeding germplasm. This study also substantiated the importance of underutilized legume species (e.g., grass pea) as potential sources of valuable protein for humans and animals. Although studies regarding the legumes’ protein quality, including underutilized grain legumes, are still scarce, the present work clearly showed the highest protein average content and range values in LS and the lowest average content in CA. LC stood out as the species with the lowest range of protein content, although it had the second-highest protein average value. PS and VF showed intermediate protein contents and range values.

A clear separation of the five legume species and respective accessions within each species under study was based on the protein content and quality, namely in the Glu and Thr contents. Indeed, protein contents, range values, and amino acid contents varied in a species-specific way among the studied collections of grain legumes and their few studied crop wild relatives. When comparing the species under study, VF and LS were the two species with the most similar amino acids and protein content. PS was the species with the most differentiated amino acid profiles and protein content. Overall, LC showed the highest amino acid average contents, and PS had the lowest average values. Nevertheless, in relation to the amino acids content range of variation, no clear species-specific trend was found in the analyzed germplasm collections, and the higher range was determined for the most abundant amino acids and the lower range for the less abundant amino acids.

The pairwise correlation analysis for the different legume species indicated positive correlations between individual amino acids but not between individual amino acids and total protein content, determined by NIR. The positive correlations between individual amino acid content were supported by the amino acids’ chemical nature and by shared amino acids’ metabolic pathways. Overall, CA and LC were the species with the highest amino acid score and protein efficiency ratio, clearly highlighting the quality of these legume species to supply human and animal amino acid requirements. LC, together with CA, were the species with the highest rate of functional amino acid residues with potential beneficial health effects (e.g., His, Lys, Pro and Met, and Tyr).

Despite the reduced/moderate protein digestibility of the studied raw legume species (less than 75%), it is worth mentioning that LC, followed by PS and LS, were the legume species with the highest IVPD values. These legume species with higher IVPD in the raw flour represent nutritious options for the feed industry that normally uses the raw materials without any complex or expensive technological processing and needs to deal with the difficult challenge of adjusting the protein supply to better meet the animals’ physiological requirements.

Taking advantage of the intra- and inter-species variability described in the present manuscript, plant breeders can improve, within each species, not only the legumes’ protein content but also the legumes’ protein quality, developing higher-quality varieties that respond more efficiently to the challenge of producing sustainable protein for all.

## Figures and Tables

**Table 1:** Calculated protein quality, amino acid scores (AAS) considering the amino acid requirements of children between 2–5 years old in mg/16 g N [20] and protein efficiency ratio (PER), in vitro protein digestibility (IVPD), %, and in vitro protein digestibility corrected amino acid score (IVPDCAAS) determined for the different legume species.

	CA (*n* = 86)	PS (*n* = 118)	VF (*n* = 92)	LC (*n* = 92)	LS (*n* = 109)
AAS_Val	143.85 ± 14.41 ^b^	112.04 ± 22.41 ^d^	123.13 ± 12.97 ^c^	165.87 ± 21.59 ^a^	117.12 ± 12.60 ^d^
AAS_Thr	96.43± 17.56 ^a^	38.46± 8.62 ^d^	75.96± 12.37 ^b^	61.76± 14.12 ^c^	35.33± 19.04 ^d^
AAS_Ile	168.07 ± 18.20 ^a^	100.62 ± 19.22 ^d^	129.99 ± 11.20 ^c^	142.19 ± 17.89 ^b^	127.02 ± 14.08 ^c^
AAS_Leu	114.96 ± 9.96 ^a^	81.64 ± 14.04 ^e^	94.97 ± 7.66 ^c^	109.63 ± 14.32 ^b^	89.47 ± 8.42 ^d^
AAS_Met	32.37 ± 3.48 ^a^	17.09 ± 2.25 ^cd^	17.64 ± 1.75 ^c^	20.61 ± 3.40 ^b^	16.37 ± 1.89 ^d^
AAS_His	119.56 ± 9.84 ^a^	57.52 ± 9.47 ^c^	101.12 ± 8.69 ^b^	119.05 ± 16.08 ^a^	99.72 ± 11.23 ^b^
AAS_Phe + Tyr	127.00 ± 10.35 ^a^	89.55 ± 17.12 ^c^	92.51 ± 7.53 ^c^	112.15 ± 13.46 ^b^	93.93 ± 9.57 ^c^
AAS_Lys	115.41 ± 9.98 ^b^	106.00 ± 15.52 ^c^	94.68 ± 8.62 ^d^	126.04 ± 17.17 ^a^	93.31 ± 8.82 ^d^
PER1	2.59 ± 0.28 ^a^	1.62 ± 0.39 ^e^	2.02 ± 0.22 ^c^	2.42 ± 0.41 ^b^	1.85 ± 0.24 ^d^
PER2	2.69 ± 0.28 ^a^	1.74 ± 0.38 ^e^	2.12 ± 0.21 ^c^	2.53 ± 0.40 ^b^	1.97 ± 0.23 ^d^
PER3	2.39 ± 0.38 ^a^	0.69 ± 0.44 ^e^	1.36 ± 0.28 ^c^	1.96 ± 0.54 ^b^	1.18 ± 0.28 ^d^
IVPD (%)	71.04± 0.38 ^ab^	72.14± 0.31 ^ab^	70.54± 1.19 ^b^	73.13± 0.14 ^a^	71.91± 0.12 ^ab^
IVPDCAAS	22.48± 0.98 ^a^	12.35± 0.24 ^c^	11.16± 0.96 ^c^	16.29± 0.67 ^b^	11.93± 0.50 ^c^

*Cicer arietinum,* CA, *Pisum sativum* and their crop wild relatives, PS, *Vicia faba*, VF, *Lens culinaris*, LC, *Lathyrus sativus* and their crop wild relatives, LS, average ± standard deviation (SD). Different letters per line indicate significant differences (*p* < 0.05) between the legume species.

## Data Availability

The data presented in this study are available within the article or Supplementary Material here.

## Supplementary Materials

The following supporting information can be downloaded at: https://www.mdpi.com/article/10.3390/foods12071383/s1, Table S1. Germplasm identification/ name of the analyzed samples and corresponding origin country.; Table S2. Amino acids content, average ± standard deviation, expressed as g/100 g (g/16 g N); % Error for g/100 g (g/16 g N). Ratio non-essential amino acids: essential amino acids (NEAAS: EAAS); % Error. Protein content, average ± standard deviation, g/100 g sample; % Error, in the different grain legume species; Table S3. Pearson correlation coefficients between 17 traits measured in five different legume species and the first two principal components (PC) scores (PC1 and PC2); Table S4. Identification of the different *Lathyrus sativus*, LS, samples in the two suggested subgroups of LS samples (above a loading score of 1.18 in PC2 – upper subgroup in the PCA score plot and below 1.18 in PC2 – lower subgroup in the PCA score plot) highlighted by principal component analysis (PCA).
