# Supplementary material for: High Inter- and Intra- Diversity of Amino Acid Content and Protein Digestibility Disclosed in Five Cool Season Legume Species with a Growing Market Demand
Source: Foods. 2023 Mar 24;12(7):1383. doi: 10.3390/foods12071383 (PMC10093753; doi:10.3390/foods12071383)
Supplement: Supplementary file 1 [file foods-12-01383-s001.zip › foods-2261834-supplementary.pdf]

Supplementary data

Table S1. Germplasm identification/ name of the analyzed samples and corresponding origin country.

Legume Species	Germplasm ID/Name	Wild/ Landrace/ Cultivar/ Breeding line/ Commercial variety [1]	Origin (Country/Institution)
Cicer arietinum	CA66	Landrace	Spain
	CA1348	Landrace	Mexico
	CA1443	Landrace	Greece
	CA1449	Landrace	India
	CA1473	Landrace	India
	CA1475	Landrace	Spain
	CA1540	Landrace	Spain
	CA1545	Landrace	India
	CA1596	Landrace	Hungary
	CA1745	Landrace	Iran
	CA1775	Landrace	Morocco
	CA1777	Landrace	Uganda
	CA1796	Landrace	Spain
	CA1800	Landrace	Jordan
	CA1821	Cultivar	India
	CA1850	Cultivar	India
	CA1889	Landrace	Morocco
	CA1935	Landrace	Spain
	CA1937	NA	Spain
	CA1938	Breeding line	Spain
	CA2139	Landrace	Spain
	CA2156	Landrace	Spain
	CA2222	Landrace	Turkey
	CA2225	Landrace	Afghanistan
	CA2235	Landrace	Russia
	CA2238	Landrace	Spain
	CA2247	Landrace	Spain
	CA2285	Landrace	Spain
	CA2297	Breeding line	ICARDA
	CA2374	Breeding line	ICARDA
	CA2379	Breeding line	ICARDA
	CA2954	Breeding line	Spain
	CA2982	Breeding line	Spain
	CA2991	Breeding line	Spain
	CA2999	Breeding line	Spain
	CA3006	Breeding line	Spain
	CA3009	Breeding line	Spain
	CA3015	Breeding line	Spain
	CA3020	Breeding line	Spain
	CA3024	Breeding line	Spain
	CA3027	Breeding line	Spain
	CA3029	Breeding line	Spain
	CA3039	Breeding line	Spain
	CA3040	Breeding line	Spain
	CA3043	Breeding line	Spain
	CA3061	Breeding line	Spain
	CA3063	Breeding line	Spain
	CA3064	Breeding line	Spain
	CA3070	Landrace	Argentina
	CA3071	Landrace	Argentina
	CA3072	Landrace	Argentina
	CA3073	Landrace	Argentina
	CA3074	Landrace	Argentina
	CA3077	Breeding line	Spain
	CA3078	Breeding line	Spain
	CA3079	Breeding line	Spain
	CA3080	Breeding line	Spain
	CA3081	Breeding line	Spain
	CA3083	Breeding line	Spain

Table S1. *Cont.*

Legume Species	Germplasm ID/Name	Wild/ Landrace/ Cultivar/ Breeding line/ Commercial variety [1]	Origin (Country/ Institution)
Cicer arietinum	CA3085	Breeding line	Spain
	CA3086	Breeding line	Spain
	CA3091	Breeding line	Spain
	CA3093	Breeding line	Spain
	CA3094	Breeding line	Spain
	CA3096	Breeding line	Spain
	CA3098	Breeding line	Spain
	CA3101	Breeding line	Spain
	AS18	Breeding line	Spain
	Garbana	Commercial variety	Spain/France
	Analisto	Commercial variety	Spain/France
	ICC6098	Breeding line	Spain
	ICC7537	Breeding line	Ethiopia
	ICCL81001	Breeding line	Spain
	ICCV88516	Breeding line	Spain
	ILC3279	Breeding line	Spain
	JG62	Breeding line	Spain
	P2245	Breeding line	Spain
	PM233	Breeding line	Spain
	PV-1	Breeding line	Spain
	RIL33	Breeding line	Spain
	WR35	Breeding line	Spain
	Fardón	Commercial variety	Spain
	Juano	Commercial variety	Spain
	Patio	Commercial variety	Spain
	Pringao	Commercial variety	Spain
	Saborio	Commercial variety	Spain
Lens culinaris	BGE996	Landrace	Spain
	BGE1023	Landrace	Spain
	BGE1048	Landrace	Spain
	BGE1055	Landrace	Spain
	BGE1080	Landrace	Spain
	BGE1087	Landrace	Spain
	BGE1140	Landrace	Spain
	BGE1378	Landrace	Spain
	BGE1394	Landrace	Spain
	BGE1409	Landrace	Spain
	BGE1415	Landrace	Spain
	BGE1430	Landrace	Spain
	BGE1431	Landrace	Spain
	BGE1443	Landrace	Spain
	BGE1459	Landrace	Spain
	BGE1491	Landrace	Spain
	BGE1805	Landrace	Spain
	BGE1808	Landrace	Spain
	BGE1823	Landrace	Spain
	BGE1824	Landrace	Spain
	BGE1835	Landrace	Spain
	BGE1845	Landrace	Spain
	BGE1846	Landrace	Spain
	BGE1850	Landrace	Spain
	BGE1867	Landrace	Spain
	BGE1868	Landrace	Spain
	BGE1880	Landrace	Spain
	BGE1885	Landrace	Spain
	BGE1892	Landrace	Spain
	BGE1908	Landrace	Spain
	BGE4246	Landrace	Spain
	BGE4247	Landrace	Spain
	BGE4248	Landrace	Spain

Table S1. *Cont.*

Legume Species	Germplasm ID/Name	Wild/ Landrace/ Cultivar/ Breeding line/ Commercial variety [1]	Origin (Country/ Institution)
	BGE4250	Landrace	Spain
	BGE4251	Landrace	Spain
	BGE8688	Landrace	Spain
	BGE8691	Landrace	Spain
	BGE8694	Landrace	Spain
	BGE8698	Landrace	Spain
	BGE11075	Landrace	Spain
	BGE11077	Landrace	Spain
	BGE11082	Landrace	Spain
	BGE11089	Landrace	Spain
	BGE11094	Landrace	Spain
	BGE11095	Landrace	Spain
	BGE13999	Landrace	Spain
	BGE16344	Landrace	Spain
	BGE16345	Landrace	Spain
	BGE16352	Landrace	Spain
	BGE16353	Landrace	Spain
	BGE16354	Landrace	Spain
	BGE16355	Landrace	Spain
	BGE16357	Landrace	Spain
	BGE16358	Landrace	Spain
	BGE16359	Landrace	Spain
	BGE19696	Landrace	Spain
	BGE19698	Landrace	Spain
	BGE19699	Landrace	Spain
	BGE19700	Landrace	Spain
	BGE19701	Landrace	Spain
	BGE19729	Landrace	Spain
	BGE19733	Landrace	Spain
Lens culinaris	BGE22153	Landrace	Spain
	BGE22526	Landrace	Spain
	BGE23248	Landrace	Spain
	BGE23249	Landrace	Spain
	BGE23250	Landrace	Spain
	BGE23655	Landrace	Spain
	BGE24339	Landrace	Spain
	BGE25599	Landrace	Spain
	BGE25600	Landrace	Spain
	BGE26701	Landrace	Spain
	BGE29091	Landrace	Spain
	BGE29092	Landrace	Spain
	BGE29684	Landrace	Spain
	BGE31048	Landrace	Spain
	BGE31052	Landrace	Spain
	BGE31056	Landrace	Spain
	BGE31057	Landrace	Spain
	BGE31058	Landrace	Spain
	BGE31063	Landrace	Spain
	BGE31070	Landrace	Spain
	BGE32284	Landrace	Spain
	BGE34196	Landrace	Spain
	BGE37770	Landrace	Spain
	BGE39490	Landrace	Spain
	BGE40543	Landrace	Spain
	BGE40544	Landrace	Spain
	BGE40545	Landrace	Spain
	BGE40546	Landrace	Spain
	BGE40547	Landrace	Spain
	BGE40549	Landrace	Spain

Table S1. *Cont.*

Legume Species	Germplasm ID/Name	Wild/ Landrace/ Cultivar/ Breeding line/ Commercial variety [1]	Origin (Country/ Institution)
	PI163293	Cultivar	India
	PI165528	Cultivar	India
	PI170469	Cultivar	Turkey
	PI170470	Cultivar	Turkey
	PI172930	Cultivar	Turkey
	PI179939	Cultivar	India
	PI180848	Cultivar	Turkey
	PI193544	Cultivar	Ethiopia
	PI195603	Landrace	Ethiopia
	PI195993	Landrace	Ethiopia
	PI195998	Landrace	Ethiopia
	PI196001	Landrace	Ethiopia
	PI220176	Cultivar	Afghanistan
	PI221467	Cultivar	Afghanistan
	PI223269	Cultivar	Afghanistan
	PI226948	Landrace	Ethiopia
	PI227847	Landrace	Iran
	PI230345	Cultivar	Iran
	PI232923	Cultivar	Hungary
	PI244756	Landrace	Ethiopia
	PI251413	Cultivar	Iran
	PI257589	Cultivar	Ethiopia
	PI268478	Cultivar	Afghanistan
	PI269921	Landrace	Pakistan
	PI283547	Landrace	France
	PI283550	Landrace	Former Soviet Union
	PI283553	Landrace	Italy
	PI283554	Landrace	Former Soviet Union
Lathyrus sativus	PI283560	Landrace	Morocco
	PI283561	Landrace	Greece
	PI283564	Landrace	Sudan
	PI283565	Landrace	Morocco
	PI283566	Landrace	Morocco
	PI283568	Landrace	Hungary
	PI283569	Landrace	Libya
	PI283570	Cultivar	Algeria
	PI283572	Landrace	Cyprus
	PI283580	Landrace	Cyprus
	PI283582	Landrace	Italy
	PI283583	Landrace	Italy
	PI283586	Landrace	Cyprus
	PI283592	Landrace	Cyprus
	PI283593	Landrace	Former Czechoslovakia
	PI283595	Landrace	Poland
	PI283596	Landrace	Afghanistan
	PI283597	Landrace	Tunisia
	PI286531	Landrace	India
	PI317440	Cultivar	Afghanistan
	PI317443	Cultivar	Afghanistan
	PI358600	Landrace	Ethiopia
	PI358601	Landrace	Ethiopia
	PI370600	Cultivar	Former Yugoslavia
	PI380888	Landrace	Iran
	PI391430	Landrace	India
	PI391431	Landrace	India
	PI391432	Landrace	India
	PI422521	Landrace	Hungary

Table S1. *Cont.*

Legume Species	Germplasm ID/Name	Wild/ Landrace/ Cultivar/ Breeding line/ Commercial variety [1]	Origin (Country/ Institution)
Lathyrus sativus	PI422526	Landrace	Hungary
	PI422532	Cultivar	Former Soviet Union
	PI422533	Landrace	Former Soviet Union
	PI422535	Landrace	Turkey
	PI422536	Landrace	Italy
	PI422537	Landrace	Hungary
	PI422538	Landrace	Former Soviet Union
	PI422540	Landrace	Italy
	PI422541	Landrace	Former Soviet Union
	PI426880	Landrace	Pakistan
	PI426884	Landrace	Pakistan
	PI426886	Landrace	Pakistan
	PI426890	Landrace	Pakistan
	PI426894	Landrace	Pakistan
	PI426897	Landrace	Pakistan
	PI442793	Landrace	India
	PI513244	Cultivar	Pakistan
	PI543071	Cultivar	Pakistan
	PI568190	Landrace	Turkey
	PI568195	Landrace	Turkey
	PI577138	Cultivar	Bulgaria
	PI577139	Landrace	Bulgaria
	PI577141	Landrace	Nepal
	PI667238	Cultivar	Greece
	PI667247	Landrace	Pakistan
	PI667250	Landrace	Albania
	PI667251	Landrace	Poland
	PI667252	Landrace	Tajikistan
	PI667263	Landrace	Georgia
	ACC170	Breeding line	ICARDA
	ACC190	Breeding line	ICARDA
	ACC192	Breeding line	ICARDA
	ACC273	Breeding line	ICARDA
	BGE1490-1	Landrace	Spain
	BGE1490-2	Landrace	Spain
	BGE1490-3	Landrace	Spain
	BGE1490-2-2-3	Landrace	Spain
	BGE15746-1	Landrace	Spain
	BGE15746-2	Landrace	Spain
	BGE15746-1-1	Landrace	Spain
	BGE17184	Landrace	Spain
	BGE23542 (*Lathyrus cicera*)	Crop Wild Relative	Spain
	BGE24709	Landrace	Spain
	BGE29748	Landrace	Spain
	BGE8277 (*Lathyrus cicera*)	Crop Wild Relative	Spain
	Lisa	Breeding line	Spain
	LS87124	Breeding line	Canada
	Raipur	Breeding line	India
	GRÃO DA COMENDA (*Lathyrus cicera*)	Commercial variety/Crop Wild Relative	Portugal
	RHODOS	Commercial variety	Greece
	SITNICA	Commercial variety	Former Yugoslavia
	STUDENICA	Commercial variety	Former Yugoslavia
Pisum sativum	PI109865	Landrace	Venezuela
	PI116056	Landrace	India
	PI122442	Commercial variety	Peru
	PI142774	Landrace	Mexico

Table S1. *Cont.*

Legume Species	Germplasm ID/Name	Wild/ Landrace/ Cultivar/ Breeding line/ Commercial variety [1]	Origin (Country/ Institution)
	PI143483	Landrace	Iran
	PI143486	Landrace	Iran
	PI162692	Cultivar	Argentina
	PI162910	Landrace	Paraguay
	PI164568	Landrace	India
	PI180329	Landrace	India
	PI184131	Landrace	Former Yugoslavia
	PI195405	Landrace	Guatemala
	PI203065	Landrace	Finland
	PI204667	Landrace	The Netherland
	PI220673	Landrace	Afghanistan
	PI234262	Landrace	USA
	PI254625	Landrace	Finland
	PI254626	Landrace	Australia
	PI262189	Landrace	Costa Rica
	PI266070	Landrace	Sweden
	PI269760	Landrace	UK
	PI272143	Landrace	Germany
	PI274584	Landrace	Norway
	PI280621	Landrace	Former Soviet Union
	PI311112	Landrace	Guatemala
	PI312135	Landrace	Guatemala
	PI314796	NA	Australia
	PI319373	Landrace	Mexico
	PI324705	NA	France
	PI326194	Landrace	Mexico
	PI343962	Wild	Turkey
	PI343984	Landrace	Turkey
	PI343993	Landrace	Turkey
	PI347316	Landrace	India
	PI347319	Landrace	India
Pisum sativum	PI347321	Landrace	India
	PI347342	Landrace	India
	PI347348	Landrace	India
	PI347366	Landrace	India
	PI347374	Landrace	India
	PI347375	Landrace	India
	PI347388	Landrace	India
	PI347389	Landrace	India
	PI347401	Landrace	India
	PI358608 (*P. abyssinicum*)	Crop Wild Relative	Ethiopia
	PI399129	Landrace	Ethiopia
	PI494079	Landrace	Chile
	PI608038	Cultivar	USA
	PI613100	Cultivar	USA
	CGN16640	Landrace	Sudan
	CGN16639	Landrace	Ethiopia
	CGN16582	Landrace	Nepal
	CGN03190	Landrace	Turkey
	CGN03165	Landrace	Turkey
	CGN03229	Landrace	Ethiopia
	IAS-3091	Landrace	Afghanistan
	Rondo	Commercial variety	Spain/Hungary/Italy
	Erygel	Commercial variety	France
	Erylis	Commercial variety	NA
	Marlin	Commercial variety	NA
	Mexique 4	Commercial variety	Mexico
	Almota	Commercial variety	NA
	Almires	Commercial variety	NA
	PI391630	Landrace	China
	IAS-2997	Breeding line	NA
	IAS-2995	Breeding line	Peru
	Atc-4235-53	Breeding line	Australia

Table S1. *Cont.*

Legume Species	Germplasm ID/Name	Wild/ Landrace/ Cultivar/ Breeding line/ Commercial variety [1]	Origin (Country/ Institution)
Pisum sativum	Attika	Commercial variety	European
	Ballet	Commercial variety	France
	Bohartyr	Commercial variety	NA
	Bonzer	Commercial variety	Australia
	Boreen	Commercial variety	Australia
	Cameor	Commercial variety	France
	IAS-3073	Breeding line (mangetout type)	Czech Republic
	Danclale	Commercial variety	Australia
	Enduro	Commercial variety	France/Spain
	Eritreo	Breeding line	Spain
	Excell	Commercial variety	Australia
	Fandango	Breeding line	Spain
	Franklin	Commercial variety	USA
	Isard	Commercial variety	France
	Kagpa	Commercial variety	Australia
	Kaspa	Commercial variety	Australia
	Lifter	Commercial variety	USA
	M5	Breeding line	Australia
	Malci	Commercial variety	Australia
	Messire	Commercial variety	France
	Moonlizht	Commercial variety	Australia
	Morris	Commercial variety	Czech Republic
	Parvus	Commercial variety	Former Czechoslovakia
	Pinochio	Commercial variety	Denmark
	Radley	Commercial variety	NA
	Toro	Breeding line	Spain
	AGT205.21	Breeding line	Czech Republic
	AGT208.5	Breeding line	Czech Republic
	AGT209.1	Breeding line	Czech Republic
	AGT209.27	Breeding line	Czech Republic
	AGT211.4	Breeding line	Czech Republic
	B99-106	Breeding line	Czech Republic
	B99-114	Breeding line	Czech Republic
	IAS-P646 (*P. sativum* ssp. *arvense*)	Crop Wild Relative	Ethiopia
	IAS-P614 (*P. sativum* ssp. *elatius*)	Crop Wild Relative	Turkey
	mfn1-1	Breeding line	NA
	mfn2-1	Breeding line	NA
	mfn1-1xmfn2-1	Breeding line	NA
	P651 (*P. fulvum*)	Crop Wild Relative	Syria
	P660 (*P. fulvum*)	Crop Wild Relative	Syria
	P665 (*P. sativum* ssp. *syriacum*)	Crop Wild Relative	Spain
	Pa316 (*P. sativum* ssp. *arvense*)	Crop Wild Relative	NA
	Pa637 (*P. sativum* ssp. *arvense*)	Crop Wild Relative	NA
	Dark Skin Perfection	Commercial variety	NA
	New Era	Commercial variety	NA
	New Season	Commercial variety	NA
	WSU28	Breeding line	USA
	WSU23	Breeding line	USA
	WSU31	Breeding line	USA
	1123	Breeding line	NA
	28805	Commercial variety	Australia
Vicia faba	BG-123	Landrace	Spain
	BG-143	Landrace	Spain
	BG-147	Landrace	Spain
	BG-153	Landrace	France
	BG-239	Landrace	India
	BG-255	Commercial variety	NA

Table S1. *Cont.*

Legume Species	Germplasm ID/Name	Wild/ Landrace/ Cultivar/ Breeding line/ Commercial variety [1]	Origin (Country/ Institution)
	BG-257	Commercial variety	NA
	BG-265	Landrace	China
	BG-266	Landrace	Ethiopia
	BG-267	Landrace	Ethiopia
	BG-269	Landrace	Ethiopia
	BG-285	Landrace	Bulgaria
	BG-288	Landrace	Bulgaria
	BG-290	Landrace	Bulgaria
	BG-291	Landrace	Bulgaria
	BG-335	Landrace	Russia
	BG-368	Landrace	Jordan
	BG-429	Landrace	Morocco
	BG-442	Landrace	Turkey
	BG-448	Landrace	Turkey
	BG-483	Landrace	Lebanon
	BG-511	Landrace	Egypt
	BG-545	Landrace	Tunisia
	BG-906	Landrace	Spain
	BG-917	Landrace	Spain
	BG-922	Landrace	Spain
	BG-927	Landrace	Spain
	BG-958	Landrace	Spain
	BG-977	Landrace	Spain
	BG-981	Landrace	Spain
	BG-992	Landrace	Spain
	BG-996	Landrace	Spain
	BG-1012	Landrace	Spain
	BG-1020	Landrace	Spain
	BG-1021	Landrace	Spain
Vicia faba	BG-1022	Landrace	Spain
	BG-1024	Landrace	Spain
	BG-1032	Landrace	Spain
	BG-1036	Landrace	Spain
	BG-1044	Landrace	Spain
	BG-1047	Landrace	Spain
	BG-1061	Landrace	Peru
	BG-1063	Landrace	Peru
	BG-1065	Landrace	NA
	BG-1067	Landrace	Iran
	BG-1068	Landrace	Afghanistan
	BG-1073	Landrace	Spain
	BG-1074	Landrace	Spain
	BG-1085	Landrace	Spain
	BG-1087	Landrace	Spain
	BG-1093	Landrace	Spain
	BG-1109	Landrace	Spain
	BG-1116	Landrace	Spain
	BG-1117	Landrace	Spain
	BG-1134	Landrace	Spain
	BG-1162	Landrace	Spain
	BG-1201	Landrace	Spain
	BG-1268	Commercial variety	NA
	BG-1295	Landrace	Mexico
	BG-1305	Landrace	NA
	BG-1375	Landrace	ICARDA
	BG-1542	Landrace	NA
	BG-1631	Landrace	CEE
	BG-1809	Landrace	Sudan
	BPL710	Breeding line	ICARDA
	ALAMEDA	Commercial variety	Spain

Table S1. *Cont.*

Legume Species	Germplasm ID/Name	Wild/ Landrace/ Cultivar/ Breeding line/ Commercial variety [1]	Origin (Country/ Institution)
Vicia faba	BARACA	Commercial variety	Spain
	JOYA	Commercial variety	Spain
	NAVIO	Breeding Line	Spain
	OMEYA	Commercial variety	Spain
	PROTHABON	Commercial variety	Spain
	QUIJOTE	Breeding Line	Spain
	PRT005-2193	Landrace	Portugal
	PRT005-2194	Landrace	Portugal
	PRT005-2219	Landrace	Portugal
	PRT005-2231	Landrace	Portugal
	PRT005-2243	Landrace	Portugal
	PRT005-2282	Landrace	Portugal
	PRT005-2288	Landrace	Portugal
	PRT005-2290	Landrace	Portugal
	PRT005-2296	Landrace	Portugal
	PRT005-2302	Landrace	Portugal
	PRT005-2304	Landrace	Portugal
	PRT005-5330	Landrace	Portugal
	PRT005-5333	Landrace	Portugal
	PRT005-5334	Landrace	Portugal
	PRT005-5357	Landrace	Portugal
	PRT005-5362	Landrace	Portugal
	VF6	Breeding line	Spain
	VF27	Breeding line	Spain
	VF136	Breeding line	Spain
	29H	Breeding line	France

1. Biological status: 1) Crop Wild Relative or native undomesticated population; 2) Landrace or traditional cultivar; 3) Breeding line or research material; 4) commercial variety. NA – information not available.

Table S2. Amino acids content, average ± standard deviation, expressed as g/100 g (g/16 g N); % Error for g/100 g (g/16 g N). Ratio non-essential amino acids: essential amino acids (NEAAS: EAAS); % Error. Protein content, average ± standard deviation, g/100 g sample; % Error, in the different grain legume species

		CA (n=86)	PS (n=118)[1]	VF (n=92)	LC (n=92)	LS (n=109)[2]
Essential amino acids (EAAs)	**Met**	0.15 ± 0.02^{a} (0.81 ± 0.09); 12.8 (10.8)	0.10 ± 0.01 ^{e} (0.43 ± 0.06); 14.3 (13.2)	0.11 ± 0.01^{d} (0.44 ± 0.04); 10.6 (9.9)	0.13 ± 0.02 ^{b} (0.52 ± 0.08); 18.4 (16.5)	0.12 ± 0.01^{c} (0.41 ± 0.05); 11.7 (11.5)
	**His**	0.43 ± 0.05 ^{d} (2.27 ± 0.19); 11.1 (8.2)	0.24 ± 0.05 ^{e} (1.09 ± 0.18); 19.3 (16.5)	0.46 ± 0.05 ^{c} (1.92 ± 0.17); 10.7 (8.6)	0.59 ± 0.09 ^{a} (2.26 ± 0.31); 14.6 (13.5)	0.53 ± 0.06 ^{b} (1.89 ± 0.21); 11.8 (11.3)
	**Thr**	0.62± 0.10 ^{a} (3.28 ± 0.60); 16.5 (18.2)	0.29± 0.06 ^{c} (1.31 ± 0.29); 22.0 (22.4)	0.62± 0.10 ^{a} (2.58 ± 0.42); 16.8 (16.3)	0.54± 0.13 ^{b} (2.10 ± 0.48); 23.2 (22.9)	0.34± 0.17 ^{c} (1.20 ± 0.65); 52.0 (53.9)
	**Ile**	0.89± 0.11 ^{c} (4.71 ± 0.51); 12.5 (10.8)	0.63± 0.14 ^{d} (2.82 ± 0.54); 22.0 (19.1)	0.87± 0.09 ^{c} (3.64 ± 0.31); 10.3 (8.6)	1.03 ± 0.14 ^{a} (3.98 ± 0.50); 14.0 (12.6)	1.00 ± 0.11 ^{b} (3.56 ± 0.39); 10.7 (11.1)
	**Leu**	1.44± 0.15 ^{c} (7.59 ± 0.66); 10.5 (8.7)	1.21 ± 0.24 ^{d} (5.39 ± 0.93); 20.0 (17.2)	1.50 ± 0.15 ^{c} (6.27 ± 0.51); 9.8 (8.1)	1.87 ± 0.26 ^{a} (7.24 ± 0.95); 14.1 (13.1)	1.66 ± 0.16 ^{b} (5.90 ± 0.56); 9.7 (9.4)
	**Phe**	1.01± 0.12 ^{b} (5.31 ± 0.46); 12.1 (8.6)	0.76 ± 0.18 ^{d} (3.41 ± 0.69); 23.2 (20.3)	0.81 ± 0.08 ^{c} (3.38 ± 0.29); 10.0 (8.6)	1.13 ± 0.15 ^{a} (4.34 ± 0.52); 13.3 (11.9)	1.01 ± 0.10 ^{b} (3.60 ± 0.36); 10.0 (10.1)
	**Val**	0.96 ± 0.10 ^{d} (5.03 ± 0.50); 10.4 (10.0)	0.88 ± 0.20 ^{e} (3.92 ± 0.78); 22.5 (20.0)	1.03 ± 0.12 ^{c} (4.31 ± 0.45); 12.0 (10.5)	1.50 ± 0.21 ^{a} (5.81 ± 0.76); 14.3 (13.0)	1.15 ± 0.12 ^{b} (4.10 ± 0.44); 10.1 (10.8)
	**Lys**	1.27± 0.11 ^{d} (6.69 ± 0.58); 9.0 (8.7)	1.37 ± 0.22 ^{c} (6.15 ± 0.90); 15.8 (14.6)	1.31 ± 0.13 ^{cd} (5.49 ± 0.50); 9.7 (9.1)	1.89 ± 0.27 ^{a} (7.31 ± 1.00); 14.2 (13.6)	1.52 ± 0.14 ^{b} (5.41 ± 0.51); 9.1 (9.5)
	**Total**	6.78 ± 0.67 ^{c} (35.69 ± 3.07); 9.9 (8.6)	5.51 ± 1.08 ^{d} (24.51 ± 4.23); 19.5 (17.3)	6.70 ± 0.65 ^{c} (28.03 ± 2.33); 9.7 (8.3)	8.83 ± 1.10 ^{a} (33.55 ± 4.28); 12.4 (12.8)	7.32 ± 0.68 ^{b} (26.08 ± 2.56); 9.3 (9.8)
Non-Essential amino acids (NEAAs)	**Tyr**	0.51 ± 0.05 ^{d} (2.69 ± 0.25); 9.5 (9.2)	0.50 ± 0.10 ^{d} (2.24 ± 0.40); 20.7 (17.9)	0.59 ± 0.06 ^{c} (2.45 ± 0.20); 10.0 (8.1)	0.70 ± 0.10 ^{a} (2.72 ± 0.36); 14.6 (13.3)	0.65 ± 0.07 ^{b} (2.32 ± 0.26); 11.5 (11.2)
	**Ser**	0.74 ± 0.08 ^{b} (3.90 ± 0.42); 10.6 (10.8)	0.61 ± 0.13 ^{c} (2.73 ± 0.54); 21.5 (19.8)	0.75 ± 0.07 ^{b} (3.13 ± 0.26); 9.1 (8.4)	1.18 ± 0.19 ^{a} (4.56 ± 0.77); 16.2 (16.8)	0.71 ± 0.06 ^{b} (2.53 ± 0.25); 8.3 (10.1)
	**Ala**	0.72 ± 0.07 ^{c} (3.78 ± 0.36); 9.2 (9.5)	0.53 ± 0.08 ^{d} (2.38 ± 0.36); 15.4 (15.3)	0.70 ± 0.06 ^{c} (2.91 ± 0.23); 9.2 (8.0)	0.94 ± 0.13 ^{a} (3.64 ± 0.53); 13.8 (14.5)	0.79 ± 0.09 ^{b} (2.80 ± 0.31); 10.9 (11.2)
	**Pro**	0.76 ± 0.08 ^{c} (3.98 ± 0.36); 10.9 (9.0)	0.71 ± 0.16 ^{d} (3.18 ± 0.63); 22.9 (19.9)	0.78 ± 0.08 ^{c} (3.28 ± 0.28); 10.3 (8.4)	1.05 ± 0.14 ^{a} (4.07 ± 0.50); 13.6 (12.3)	0.93 ± 0.10 ^{b} (3.31 ± 0.36); 10.4 (10.9)
	**Gly**	1.33 ± 0.15 ^{d} (7.03 ± 0.73); 11.2 (10.4)	1.04 ± 0.35 ^{e} (4.62 ± 1.48); 33.9 (31.9)	1.61 ± 0.28 ^{b} (6.74 ± 1.10); 17.5 (16.4)	2.39 ± 0.31 ^{a} (9.24 ± 1.30); 13.2 (14.0)	1.46 ± 0.25 ^{c} (5.22 ± 0.99); 17.2 (19.0)
	**Arg**	1.49 ± 0.29 ^{c} (7.78 ± 0.92); 19.7 (11.8)	1.25 ± 0.43 ^{d} (5.51 ± 1.47); 34.5 (26.6)	1.96 ± 0.32 ^{ab} (8.14 ± 0.86); 16.4 (10.6)	1.82 ± 0.29 ^{b} (7.00 ± 0.99); 16.0 (14.2)	2.03 ± 0.28 ^{a} (7.21 ± 0.81); 13.9 (11.2)
	**Glu**	3.25 ± 0.59 ^{b} (17.08 ± 2.78); 18.3 (16.3)	1.44 ± 0.31 ^{c} (6.46 ± 1.36); 21.7 (21.0)	3.51 ± 0.59 ^{a} (14.66 ± 2.24); 16.9 (15.3)	3.13 ± 0.74 ^{b} (12.07 ± 2.72); 23.6 (22.6)	1.65 ± 0.97 ^{c} (5.92 ± 3.61); 59.1 (61.0)
	**Asp**	2.62 ± 0.30 ^{c} (13.74 ± 0.89); 11.3 (6.5)	2.30 ± 0.64 ^{d} (10.20 ± 2.36); 27.8 (23.2)	2.76 ± 0.31 ^{bc} (11.51 ± 1.03); 11.4 (8.9)	4.49 ± 0.54 ^{a} (17.34 ± 2.03); 12.0 (11.7)	2.77 ± 0.29 ^{b} (9.87 ± 1.12); 10.5 (11.3)
	**Total**	11.42 ± 1.39 ^{c} (59.99 ± 5.05); 12.1 (8.4)	8.42 ± 2.06 ^{d} (37.32 ± 7.66); 24.5 (20.5)	12.66 ± 1.57 ^{b} (52.82 ± 5.13); 12.4 (9.7)	15.96 ± 1.96 ^{a} (60.64 ± 8.06); 12.3 (13.3)	10.99 ± 1.58 ^{c} (39.18 ± 6.06); 14.4 (15.5)
**Ratio NEAAs:EAAs**		1.68 ± 0.10 ^{c}; 6.1	1.52 ± 0.10 ^{d}; 6.6	1.88 ± 0.09 ^{a}; 4.8	1.81 ± 0.07 ^{b}; 4.1	1.50 ± 0.15 ^{d}; 9.8
**Protein**		19.08 ± 2.03 ^{e}; 10.7	22.47 ± 2.63 ^{d}; 11.7	23.97 ± 1.89 ^{c}; 7.9	25.93 ± 1.90 ^{b}; 7.3	28.17 ± 1.97 ^{a}; 7.0

1. The crop wild relatives (n=8), *Pisum fulvum*, *Pisum abyssinicum*, *Pisum sativum* spp. *arvense*, *Pisum sativum* spp. *elatius*, and *Pisum sativum* spp. *syriacum*
2. The crop wild relatives (n=2), *Lathyrus cicera*

Cicer arietinum, CA, *Pisum sativum* and their crop wild relatives, PS, *Vicia faba*, VF, *Lens culinaris*, LC, *Lathyrus sativus* and their crop wild relatives, LS. For each parameter, the significant differences between the legume species were indicated by different letters, per row, $p < 0.05$

TableS3. Pearson correlation coefficients between 17 traits measured in five different legume species and the first two principal components (PC) scores (PC1 and PC2)

Trait	PC1	PC2
AlaTran	0.953 ***	−0.049 ns
Arg	0.755 ***	0.245 ***
Asp	0.909 ***	−0.100 *
Glu	0.450 ***	−0.825 ***
Gly	0.910 ***	−0.223 ***
His	0.924 ***	0.039 ns
Ile	0.926 ***	0.041 ns
Leu	0.974 ***	0.117 *
Lys	0.868 ***	0.214 ***
MetTran	0.663 ***	−0.330 ***
PheTran	0.890 ***	0.066 ns
Pro	0.956 ***	0.201 ***
Protein	0.522 ***	0.677 ***
Ser	0.876 ***	−0.210 ***
Thr	0.418 ***	−0.849 ***
Tyr	0.930 ***	0.247 ***
Val	0.960 ***	0.081 ns

*$p < 0.05$; ***$p < 0.001$; ns – not significant

Table S4. Identification of the different *Lathyrus sativus*, LS, samples in the two suggested subgroups of LS samples (above a loading score of 1.18 in PC2 – upper subgroup in the PCA score plot and below 1.18 in PC2 – lower subgroup in the PCA score plot) highlighted by principal component analysis (PCA).

Subgroup in PCA plot	Germplasm ID/ Name	Origin (Country)	Country Region	Seed colour	Seed size[1]
**Upper subgroup LS samples**	PI163293	India	South Asia	Dark	Small
	PI165528	India	South Asia	Light	Small
	PI170469	Turkey	Mediterranean	Dark	Small
	PI170470	Turkey	Mediterranean	Dark	Small
	PI172930	Turkey	Mediterranean	Dark	Small
	PI179939	India	South Asia	Dark	Small
	PI180848	Turkey	Mediterranean	Dark	Small
	PI193544	Ethiopia	Sub-Saharan Africa	Dark	Small
	PI195603	Ethiopia	Sub-Saharan Africa	Dark	Small
	PI195993	Ethiopia	Sub-Saharan Africa	Dark	Small
	PI195998	Ethiopia	Sub-Saharan Africa	Dark	Small
	PI196001	Ethiopia	Sub-Saharan Africa	Dark	Small
	PI220176	Afghanistan	South Asia	Dark	Small
	PI221467	Afghanistan	South Asia	Dark	Small
	PI223269	Afghanistan	South Asia	Dark	Small
	PI227847	Iran	South Asia	Dark	Small
	PI230345	Iran	South Asia	Dark	Small
	PI232923	Hungary	East Europe	Light	Large
	PI244756	Ethiopia	Sub-Saharan Africa	Dark	Small
	PI251413	Iran	South Asia	Dark	Small
	PI257589	Ethiopia	Sub-Saharan Africa	Dark	Small
	PI268478	Afghanistan	South Asia	Dark	Small
	PI269921	Pakistan	South Asia	Dark	Small
	PI283547	France	Mediterranean	Dark	Small
	PI283550	Former Soviet Union	North Asia	Light	Small
	PI283553	Italy	Mediterranean	Light	Small
	PI283554	Former Soviet Union	North Asia	Light	Large
	PI283560	Morocco	Mediterranean	Light	Small
	PI283561	Greece	Mediterranean	Light	Small
	PI283564	Sudan	Sub-Saharan Africa	Dark	Small
	PI283565	Morocco	Mediterranean	Dark	Small
	PI283566	Morocco	Mediterranean	Light	Small
	PI283568	Hungary	East Europe	Light	Small
	PI283569	Libya	Mediterranean	Light	Large
	PI283570	Algeria	Mediterranean	Light	Small
	PI283572	Cyprus	Mediterranean	Light	Small
	PI283580	Cyprus	Mediterranean	Light	Large
	PI283582	Italy	Mediterranean	Dark	Small
	PI283583	Italy	Mediterranean	Dark	Small
	PI283586	Cyprus	Mediterranean	Dark	Small
	PI283592	Cyprus	Mediterranean	Light	Small
	PI283593	Former Czechoslovakia	East Europe	Dark	Small
	PI283595	Poland	East Europe	Light	Small
	PI283596	Afghanistan	South Asia	Dark	Small
	PI283597	Tunisia	Mediterranean	Light	Large

Table S4. *Cont.*

Subgroup in PCA plot	Germplasm ID/ Name	Origin (Country)	Country Region	Seed colour	Seed size[1]
**Upper subgroup LS samples**	PI286531	India	South Asia	Dark	Small
	PI317440	Afghanistan	South Asia	Light	Small
	PI317443	Afghanistan	South Asia	Dark	Small
	PI358600	Ethiopia	Sub-Saharan Africa	Dark	Small
	PI358601	Ethiopia	Sub-Saharan Africa	Dark	Small
	PI370600	Former Yugoslavia	Mediterranean	Dark	NA
	PI380888	Iran	South Asia	Dark	Small
	PI391431	India	South Asia	Dark	Small
	PI391432	India	South Asia	Dark	Small
	PI422521	Hungary	East Europe	Dark	Small
	PI422526	Hungary	East Europe	Light	Large
	BGE17184	Spain	Mediterranean	Light	Large
	PI422532	Former Soviet Union	North Asia	Light	Small
	PI422535	Turkey	Mediterranean	Light	Large
	PI422536	Italy	Mediterranean	Light	Small
	PI422537	Hungary	East Europe	Light	Small
	PI422538	Former Soviet Union	North Asia	Light	Small
	PI422540	Italy	Mediterranean	Light	Small
	PI422541	Former Soviet Union	North Asia	Light	Small
	PI426880	Pakistan	South Asia	Dark	Small
	PI426884	Pakistan	South Asia	Dark	Small
	PI426886	Pakistan	South Asia	Dark	Large
	PI426890	Pakistan	South Asia	Dark	Small
	PI426894	Pakistan	South Asia	Dark	Small
	PI426897	Pakistan	South Asia	Dark	Small
	PI577138	Bulgaria	East Europe	Dark	Large
	PI577139	Bulgaria	East Europe	Light	Small
	PI577141	Nepal	South Asia	Dark	Small
	PI667238	Greece	Mediterranean	Light	Small
	ACC190	ICARDA	NA	Dark	Small
	BGE1490-1	Spain	Mediterranean	Light	Large
	BGE1490-2	Spain	Mediterranean	Light	Large
	BGE1490-3	Spain	Mediterranean	Light	Large
	BGE15746-2	Spain	Mediterranean	Light	Large
	Lisa	Spain	Mediterranean	Light	Large
**Lower subgroup LS samples**	PI226948	Ethiopia	Sub-Saharan Africa	Dark	Small
	PI391430	India	South Asia	Dark	Small
	PI442793	India	South Asia	Dark	Small
	PI667247	Pakistan	South Asia	Dark	Small
	PI667250	Albania	Mediterranean	Light	Large
	PI667251	Poland	East Europe	Light	Large
	PI667252	Tajikistan	North Asia	Dark	Small
	PI667263	Georgia	North Asia	Light	Small
	ACC192	ICARDA	NA	Dark	Small
	ACC273	ICARDA	NA	Dark	Small
	BGE1490-2-2-3	Spain	Mediterranean	Light	Large
	BGE15746-1	Spain	Mediterranean	Light	Large
	BGE15746-1-1	Spain	Mediterranean	Light	Large
	BGE23542 (*Lathyrus cicera*)	Spain	Mediterranean	NA	NA

Table S4. *Cont.*

Subgroup in PCA plot	Germplasm ID/ Name	Origin (Country)	Country Region	Seed colour	Seed size[1]
**Lower subgroup LS samples**	BGE24709	Spain	Mediterranean	Light	Large
	BGE29748	Spain	Mediterranean	Light	Large
	LS87124	Canada	North America	Light	Small
	GRÃO DA COMENDA (*Lathyrus cicera*)	Portugal	Mediterranean	Dark	NA
	RHODOS	Greece	Mediterranean	Dark	Small
	SITNICA	Former Yugoslavia	Mediterranean	Dark	Small
	STUDENICA	Former Yugoslavia	Mediterranean	Light	Large

[1]Seed size characterization was based on the weight of 100 seeds weight. Accessions with a 100 seed weight below 18g were classified as small and accessions with a 100 seed weight over 18g were classified as large. NA – information not available
